# Lack of pre-antiretroviral care and competition from traditional healers, crucial risk factors for very late initiation of antiretroviral therapy for HIV - A case-control study from eastern Uganda

**DOI:** 10.4314/pamj.v8i1.71155

**Published:** 2011-04-07

**Authors:** Lubega Muhamadi, Nazarius Mbona Tumwesigye, Daniel Kadobera, Gaetano Marrone, Fred Wabwire-Mangen, George Pariyo, Stefan Peterson, Anna Mia Ekström

**Affiliations:** 1 District Health Office, Iganga District Administration, PO Box 358, Iganga, Uganda,; 2 Department of Epidemiology and Biostatistics, Makerere University School of Public Health, PO Box 7072, Kampala, Uganda; 3 Makerere University Iganga/Mayuge Health and Demographic Surveillance System PO BOX 7072 Kampala, Uganda; 4 Division of Global Health, IHCAR, Department of Public Health Sciences Karolinska Institutet, Sweden; 5 Department of Health Policy Planning and Management, Makerere University School of Public Health, PO Box 7072, Kampala, Uganda; 6 IMCH, Department of Women’s and Children’s Health, Uppsala University, Sweden; 7 Institute of Health Sciences Busoga University, PO Box 154, Iganga, Uganda; 8 Department of Infectious Diseases, Karolinska University Hospital, Sweden

**Keywords:** Pre-antiretroviral care, competition form traditional healers, Very late ART initiation

## Abstract

**Background:**

Although WHO recommends starting antiretroviral treatment at a CD4 count of 350 cells/[µ]L, many Ugandan districts still struggle with large proportions of clients initiating ART very late at CD4 < 50 cells/[µ]L. This study seeks to establish crucial risk factors for very late ART initiation in eastern Uganda.

**Methods:**

All adult HIV-infected clients on ART in Iganga who enrolled between 2005 and 2009 were eligible for this case-control study. Clients who started ART at CD4 cell count of < 50 cells/[µ]L (very late initiators) were classified as cases and 50-200 cells/[µ]L (late initiators) as control subjects. A total of 152 cases and 202 controls were interviewed. Multivariate analyses were performed to calculate adjusted odds ratios and 95% confidence intervals.

**Results:**

Reported health system-related factors associated with very late ART initiation were stock-outs of antiretroviral drugs stock-outs (affecting 70% of the cases and none of the controls), competition from traditional/spiritual healers (AOR 7.8, 95 CI% 3.7-16.4), and lack of pre-ARV care (AOR 4.6, 95% CI: 2.3-9.3). Men were 60% more likely and subsistence farmers six times more likely (AOR 6.3, 95% CI: 3.1-13.0) to initiate ART very late. Lack of family support tripled the risk of initiating ART very late (AOR 3.3, 95% CI: 1.6-6.6).

**Conclusion:**

Policy makers should prevent ARV stock-outs though effective ARV procurement and supply chain management. New HIV clients should seek pre-ARV care for routine monitoring and determination of ART eligibility. ART services should be more affordable, accessible and user-friendly to make them more attractive than traditional healers

## Background

Very late initiation of antiretroviral therapy (ART) for people living with HIV (PLHIV) is a major concern especially at a time when WHO is advocating for earlier ART initiation at a CD4 cell count of < 350 cells/[µ]L [1-3]. Delayed initiation of ART is associated with late-stage diagnosis and results in high rates of HIV-related morbidity and mortality [4]. In sub-Saharan Africa, while the overall coverage of ART initiation has increased to 48% of those in need, emerging evidence shows that up to 59% of PLHIV are either lost to follow-up or start ART at a very late stage of the disease [5,6].

Uganda ART services have reached a reported ARV coverage of 57.5% of those eligible for treatment at a threshold for ART initiation of CD4 cell counts < 200 cells/[µ]L [7,8]. The coverage would, however, decrease to 40.4% if estimated based on the current WHO recommended threshold of < 350 cells/[µ]L[8].The Ugandan HIV/AIDS National Strategic Plan 2007/8 – 2011/12 projected an increase in ART coverage to about 80% by 2011/12 . However, limited uptake of, and delayed access to, HIV services are still a major stumbling-block to reaching these targets [9].

In Iganga district, eastern Uganda, 40% (360 out of 900) of PLHIV who initiated ART between 2004 and 2009 had a CD4 count of < 50 cells/[µ]L, with a median CD4 count of 126 (range 14-198) (DHO 2009 unpublished). Recent studies also show that 40% of PLHIV in Uganda present for HIV care late or very late at WHO disease stage 3 or 4, many with CD4 cell counts of < 50 cells/[µ]L. [6,10,11]. There is paucity of information on why PLHIV would initiate ART very late in a country where HIV and ART awareness is presumably high [9]. Subsequently, there has been a call for additional studies to determine whether late disease presentation and hence late ART initiation is due to delays in testing or accessing care [12]. The research team recently conducted a qualitative study involving clients who had initiated ART very late at a CD4 count of < 50 cells/[µ]L in Iganga district, eastern Uganda, to explore reasons for very late ART initiation. Health systems and individual/community related themes emerged as the main client reported barriers to timely initiation of ART [13]. The team therefore sought to identify crucial health system or individual/community related risk factors for very late initiation of ART in the same area. The aim was to provide information that could help policy and decision-makers in setting priorities for enhancing timely initiation of ART in Iganga district and other similar settings in Uganda and beyond.

## Methods

**Study area and study setting**

The study was conducted at Iganga District Hospital which is located 115 kilometres east of the capital Kampala. The hospital was chosen because until 2008 (most of the period under study) it was the only ART-providing unit in the district and it had the best updated ART register. ART services were first initiated at the hospital in 2004.

The district is predominantly rural with only about 7% living in a peri-urban environment, namely Iganga town. The majority of the people belong to the Bantu ethnic tribe called Basoga, most of whom depend on the subsistence farming of food crops. There are 101 health units in the district; 83 of which are owned by the government and 18 are run by non-government organisations (NGOs). District ART services are organised according to the Uganda national hierarchical referral system. Seventy (70) of the units are health centres (HC) II offering only cotrimoxazole refills and home-based care services to PLHIV; 27 are HC III offering routine counselling and testing, pre-ARV and home-based care services to PLHIV; and 3 are HC IV offering routine counselling and testing, home-based care, pre-ARV care and ART services to PLHIV. Other care providers include over 200 private drugstores/private clinics located in Iganga town and several other smaller towns scattered throughout the district. Most of the drugs commonly found in these drugstores and clinics include painkillers, assorted antibiotics and cheap antimalarials but no antiretroviral drugs (ARVS). There are no other accredited ART providers in the district, although over 100 traditional and spiritual healers are presumed by the community to offer “similar” services in the district (DHO 2009 unpublished, [14]).

Over 40 000 people (6.7% of the district adult population) currently live with HIV in Iganga district and 6 000 PLHIV are presumed to be eligible for ART [15,16]. Only 1 150 PLHIV (i.e. less than 20% of those in need), however, currently access ART. Until 2008, when two more health centres were accredited, ART services could only be accessed at the district hospital. The Iganga district ART services are occasionally managed by a medical doctor but more often by an assistant physician or a nurse. The services offered include refills of antiretroviral drugs (ARVS) and cotrimoxazole, adherence counselling, psychosocial and nutritional support once a week (DHO 2008 unpublished).

**Study design**

The study employed a case-control design for clients under ART care and was carried out between January and February 2010 at Iganga District Hospital. The case definition was HIV-infected clients who started ART with a CD4 cell count of < 50 cells/[µ]L (very late initiators). The control subjects consisted of clients who started ART with a CD4 cell count of 50-200 (late initiators) based on the current WHO recommendation for starting ART at CD4 cell count of < 350 cells/[µ]L[3].

**Study population**

All adult clients on ART in the hospital who enrolled for ART during the study period (January 2005-December 2009) were eligible for the study. Given that 40% of the clients initiated on ART in Iganga district between 2004 and 2009 had a CD4 cell count of < 50 cells/[µ]L and that the mean and median CD4 cell counts at initiation of ART for all clients were 122 and 126 cells/[µ]L respectively (range 14-198) (DHO 2009 unpublished), these margins made all clients initiated on ART during the study period eligible.

**Inclusion and exclusion criteria**

Clients on ART who had been enrolled in the ART register at the hospital and were alive and of sound mental status were included in the study. Clients who initiated ART at the hospital before 2005 were excluded to reduce the risk of bias due to the unmet demand that had to be catered for during the first months of programme initiation in 2004. Clients in the Prevention of Mother-to-Child Transmission Programme (PMTCT) were also excluded because they had already been enrolled in another study at the same site.

**Sampling size and sampling procedure**

Fleiss formula for sample size calculation was used under the following two assumptions: a) that the true ratio of cases to controls as observed from the hospital ART register was 40:60. b) that exposure to pre-ARV care was similar for both cases and control subjects (50%). Exposure to pre-ARV care was used in sample size calculation because it is a very important explanatory variable for timely entry into HIV care in the area [17,18]. Thus with a confidence interval of 95 % and power of 80%, a minimum of 124 cases and 186 controls were needed to reach statistically significant results for the study. To cater for clients who would likely withdraw consent or difficult to trace for interviews, an additional 20% margin of subjects were recruited. In total 152 cases and 243 controls were recruited for the study.

The cases and controls subjects were identified using systematic random sampling as described below: A Chart review of the ART register at the hospital for the study period was done. From the review, all clients who satisfied the selection criteria above were identified and divided into cases and control subjects in accordance with the CD4 count definitions above. For each category (case vs control), a list with all clients’ ART registration numbers, was divided into serial pairs. Using simple random sampling, one of the two first numbers of each starting serial pair for each category was selected as the first client for the study followed by every subsequent third client in each category until the desired sample size had been realised.

Trained research assistants then traced the clients for interviews using medical record information on address/place of living to seek informed consent. All the selected cases were subsequently interviewed, but 31 controls could not be interviewed either because they withdrew their consent for the interviews or could not be traced. Thus, the total number of interviewees enrolled in the study was 152 cases and 202 control subjects.

**Data collection**

Semi-structured interviewer-administered questionnaires were employed for the study. Six research assistants who had previous experience in quantitative data collection from PLHIV within the Iganga-Mayuge demographic surveillance site interviewed the clients. The research assistants were trained for four days on the study aim, design and tools. The tools were pilot-tested at the nearest HC IV offering ART. Experiences from the pilot study were discussed at an extra session together with the research assistants. Necessary changes were made to the tools and the assistants received additional guidance.

The data collected included individual/community related factors associated with timely/late initiation of ART such as the clients’ age, gender, education, occupation, religion, number of people living in the household and marital status. Other individual/community related information included; client-perceived barriers to timely ART, client perceptions and misconceptions about ARVs, CD4 count at ART initiation, presence of family support and perceived confidentiality of the staff at the ART clinic (coded as trustworthy vs not trustworthy).

Health system-related data such as client reported access to information on when to start ARVS after VCT, access to post test pre-ARV care and waiting time for CD4 cell count results was also collected, Other health system related data collected included waiting time between prescription and accessing ARVS (and if >1 month the reasons for waiting that long), clients’ distance to the ARV centre, any care sought from other providers such as traditional/spiritual healers before coming to the ARV centre and reasons for their choice of service provider. The data were checked for consistency and completeness by the first and second author (LM and TN) throughout the data collection period,

**Data management and analysis**

Data were double-entered and the two data sets were checked for discrepancies using epi-Info version 2000 (CDC Atlanta). The data were then exported to STATA8 (STATA Corporation, college station TX USA) for univariate, bivariate and multivariate analysis. Following frequency distributions and cross-tabulations, the strength of association between variables was determined using odds ratios (OR) and 95% confidence intervals (CI). For the bivariate analysis, cross-tabulations were run for each independent variable against the outcome variable (very late ART initiation). An independent variable was presumed to be significant if the cross-tabulation generated a p-value of < 0.05. Multivariate analysis was also done to control for confounding and test effect-modification. A logistic model was constructed to determine the best model for prediction of very late initiation of ART among ART clients. This process involved putting all plausible variables found to be significantly associated with very late initiation of ART among ART clients during the bivariate analysis into an initial model. The best model was thereafter generated using backward elimination and likelihood ratios to select significant variables.

**Ethical clearance**

This study was approved by the Makerere University School of Public Health Institutional Review Board, the Uganda National Council for Science and Technology and the Iganga district authorities. The respondents were informed about the study aims, their discretion to participate or withdraw at any time and assured that all information obtained from them would be kept confidential. The anticipated benefits or harm of the study to the participants or the community were clearly explained and all the study participants signed consent forms before the interviews commenced.

## Results

A total of 354 clients (152 cases initiating ART at very late a CD4 count of < 50 cells/[µ]L and 202 control subjects initiating ART at a CD4 cell count of 50-200 cells/[µ]L) were interviewed for the study. The mean age of the respondents was 38.0 years and 41.4 years for the cases and controls respectively. The majority of both cases (82%) and controls (61%) were female. More cases (80.5%) than controls (59.1%) were unmarried and

84.5% of the very late initiators were subsistence farmers compared to only 40.9% among the controls. The majority of cases (70.2%) and none of the controls reported having experienced ARV stock outs (Table 1).

Barriers to timely initiation of ART included ARV stock-outs, competition from traditional healers, inadequate pre-ARV care and lack of family support as reported by the very late initiators. Other reported barriers for timely initiation of ART were personal characteristics such as being male, being a subsistence farmer or being younger (Table 2). Very late initiators reported seeking care from traditional/spiritual healers because they perceived the healers as being accessible, nearer, less expensive, more holistic and better quality care providers than the public ART services.

**Bivariate analysis**

The bivariate analysis established that very late initiation of ART was a function of both individual/community related factors as well as important health-system related factors.

**Individual/community related factors**

Younger clients had a higher risk of initiating ART very late compared to older clients OR 0.9 (95% CI 0.8-0.9). Low educated clients were 14 times more likely to initiate ART very late compared to clients who were well-educated OR 13.5 (95% CI 7.8-23.1). Clients who were unmarried were five times more likely to initiate ART very late compared to those who were married OR 5.3 (95% CI 3.2-8.6). Subsistence farmers were eight times more likely to initiate ART very late compared to non-farmers OR 7.8 (95% CI 4.7-13.1 Clients who lacked family support were seven times more likely to initiate ART very late compared to those who had family support OR 6.7 (95% CI 4.1-11.1) (Table 2).

**Health system-related factors**

Clients who had reportedly not attended pre-ARV care were eight times more likely to initiate ART vary late compared to those who had attended pre-ARV care, OR 8.2 (95% CI 5.1-13.3). In addition, clients who had reportedly sought care from traditional/ spiritual healers before seeking formal ART care were 17 times more likely to initiate ART very late compared to those who did not seek prior traditional/spiritual care OR 17.1 (95% CI 9.1-29.6) (Table 2).

**Multivariate analysis**

The variables that remained significantly associated with very late ART initiation in the multivariate analysis included: having sought previous care from traditional/spiritual healers for AIDS related symptoms, adjusted odds ratio (AOR) 7.8 (95% CI 3.7-16.4), lack of pre-ARV care AOR 4.6 (95% CI 2.3-9.3), subsistence farming, AOR 6.3 (95% CI 3.1-13.0), lack of family support AOR 3.3 (95% CI 1.6-6.6), increase in age as a continuous variable AOR 0.9 (95% CI 0.8-0.9) and being female AOR 0.4 (95% CI 0.2-0.8), the latter two being protective against very late initiation of ART (Table 2).

## Discussion

Risks factors associated with very late initiation of ART included health system-related factors such as ARV stock-outs, competition from traditional/spiritual healers, and lack of pre-ARV care. Others were individual/community factors such as younger age, being male, being a subsistence farmer and lacking family support.

Many cases reported that they initiated ART very late because at the time of prescription, they were informed that ARVs were out of stock. Previous studies have established that timely provision of drugs improves the utilization of health services [19-26]. The ARV stock-outs could have been due to both an inefficient supply and procurement chain and/or a poor data management system that undermined forecasting for the quantities of drugs required. The stock-outs could also have been due to shortfalls in funding for procuring the drugs. The finding supports other reports on ART-eligible clients failing to access ARVS on time due to stock-outs and poor coordination of procurement procedures in Uganda that appears to be lagging far behind in terms of scale-up priorities [23,27-30].

Many very late ART initiators reported seeking care from traditional/spiritual healers before going to the centres for ART provision since they perceived the healers to be more accessible, cheaper, more holistic and providing better quality care (Figure 2). Our finding is supported by other studies in sub-Saharan Africa which have also shown that traditional/spiritual healers influence people’s health-seeking behaviour [14,31-35].

Many very late ART initiators had also not attended pre-ARV care in contrast to the late initiators. Lack of pre-ARV care could be attributed to late presentation/late diagnosis and hence failure to benefit from the advantages of early diagnosis such as post-test counselling and routine monitoring of eligibility for ART. As demonstrated also by previous research from resource-poor high prevalence settings in Uganda, South Africa and Asia, the finding highlights the importance of early HIV diagnosis, adequate post-test counselling and follow-up for PLHIV in pre-ARV care to ensure timely initiation of ART [14,17,18,36-38].

Increase in age was protective against very late initiation of ART. The finding could be attributed to increased awareness of the dangers of not seeking care for AIDS-related symptoms, reduced HIV-related stigma or the importance of seeking care related to living longer and accomplishing responsibilities as established by other studies [39].

The deliberate exclusion of women diagnosed with HIV and initiated on ART through the PMTCT programme, enabled us to look at the influence of gender in terms of seeking ART, regardless of the coverage and quality of PMTCT. We found that women were 60% less likely to start ART very late compared to men. The finding could be a reflection the even stronger stigma and denial associated with HIV seen among men in Iganga district [13]. Other studies have also shown that females have been found to seek HIV care earlier than men by other studies [40,41].

Subsistence farmers were six times more likely to start ART very late (AOR 6.3, 05% CI 3.1-13.0) compared to non-subsistence farmers. Subsistence farming is characterized by poverty and hence lack of purchasing power for social services and lack of awareness or access to vital information and communication about health care services. Poverty has been established as a predictor for late or poor health-seeking behaviour by other studies in low and medium-income countries [41-45].

Clients who lacked family support were more likely to initiate ART very late compared to those who had family support. Family support helps in accessing ART services and understanding behavioural and routine changes in the schedule of PLHIV such as monthly visits to the ART centre. Family support also enhances daily adherence to ART and discourages HIV/AIDS-related stigma as supported by previous research from Africa, Asia and South America [38,46-51].

**Methodological considerations**

The use of CD4 count as a measure for very late initiation of ART influences the interpretation of our results because a low CD4 count does not automatically translate into late disease or symptomatic disease but varies somewhat among individuals. The decision to use CD4 counts to classify case-status as well as the low cut-off margins used was made to facilitate comparisons with other studies taking the current WHO classification of late presentation as a standard. The decision was also based on our clinical observations and other studies performed in Uganda about the prognosis of very late ART initiators.

The influence of the possible selection bias introduced by only including subjects alive at the time of study, often encountered when using retrospective real-life data, is impossible to determine. Nor could recall bias be excluded but it is unlikely to have played any major role in this study since the identified risk factors did not require any detailed retrospective recall that could be assumed to differ between cases and controls subjects in this study.

Some confidence intervals are wide due to the fairly small sample size. However, the sampled data still allowed us to identify a number of statistically significant predictors for very late initiation of ART with enough precision to lay the ground for the necessary health systems and policy interventions needed to encourage earlier ART initiation. The findings of this study should be generalizable to most other rural districts in Uganda and surrounding countries with similar health system structures.

## Conclusion

In conclusion, policymakers and providers should put more emphasis on strategic ARV procurement and supply to prevent stock-outs through appropriate coordination and control of the different stakeholders at national and sub national levels. There is need to ensure that all new HIV clients access regular pre-ARV care for routine monitoring in order start ART on time. Pre-ARV care should encourage status disclosure to the immediate family to enhance social support which is key to improving access to ART [47,52]. Making services more affordable, accessible and user-friendly through peripheral units and trained, as well as supervised, lay workers is one way of making ART centres/services more attractive to potential end users than traditional healers [19,53-55].

## Competing interests

The authors declare no competing interests.

## Authors contributions

LM and TN were involved in the inception and data collection for this study. All the authors were substantively involved in the design, analysis, interpretation and manuscript revising for the study.

## Acknowledgements

This study received financial support from Sida and logistical support from the ARVMAC project funded by the European Commission. Its contents are solely the responsibility of the authors and do not reflect the views of Sidas or EU.

## Figures and Tables

**Table 1:** Univariate analysis of explanatory variables for ART initiation in Iganga, eastern Uganda, (N=354)

Characteristic	CD4 <50 (n=152)	CD4 50-200 (n=202)
	Number (%)	Number (%)
**Sex**		
Male	28 (18.4)	78 (38.7)
Female	124 (81.6)	124 (61.3)
**Mean Age**	38.0 (SD=8.8)	41.4 (SD=8.9)
**Education**		
Well educated	54 (35.5)	178 (88.1)
Low education	98 (64.5)	24 (11.9)
**Marital status**		
Married	30 (19.5)	82 (40.9)
Unmarried	122 (80.5)	120 (59.1)
**Occupation**		
Non-farmer	24 (15.8)	120 (59.1)
Subsistence farmer	128 (84.2)	82 (40.9)
**Distance to unit (mean)**	17.9 (SD=14.6)	14.6 (SD=15.2)
**ARV supplies experienced by patients**		
Stock-outs	107 (70.2)	00 (00.0)
No stock-outs	45 (29.8)	202 (100.0)
**Confidence in staff**		
Present	00 (00.0)	202 (100.0)
Absent	152 (100.0)	00 (00.0)
**Family support**		
Available	28 (18.4)	122 (60.4)
Not available	124 (81.6)	80 (39.6)
**Pre-ARV care**		
Attended	38 (25.0)	148 (73.3)
Not attended	114 (75.0)	54 (26.7)
**Traditional/Spiritual care-seeking before ART**		
No	46 (30.3)	178 (88.1)
Yes	106 (69.7)	24 (11.9)

**Table 2:** Factors associated with very late initiation (CD4 < 50) of ART in Iganga, eastern Uganda, (N=354)

Variable	Bivariate		Multivariate	
		p-value	Crude OR (95% CI)	p-value	Adjusted OR (95% CI)
**Sex**				
Male		1		1
Female	<0.001	2.8 (1.7 -4.6)	0.010	0.4 (0.2 -0.8)
**Age (continuous)**	0.001	0.9 (0.8 -0.9)	0.001	0.9 (0.8 -0.9)
**Education**				
Well educated		1		1
Low education	<0.001	13.5 (7.8 -23.1)	0.080	2.2 (0.9-5.1)
**Marital status**				
Married		1		1
Unmarried	<0.001	5.3 (3.2 -8.6)	0.120	1.8 (0.8 -3.7)
**Occupation**				
Non-farmer		1		1
Subsistence farmer	<0.001	7.8 (4.7 -13.1)	<0.001	6.3 (3.1-13.0)
**Family support**				
Available		1		1
Not available	<0.001	6.7 (4.1 -11.1)	0.001	3.3 (1.6 -6.6)
**Pre-ARV care**				
Attended		1		1
Not attended	<0.001	8.2 (5.1 -13.3)	<0.001	4.6 (2.3 -9.3)
**Traditional/Spiritual care-seeking before ART**				
No		1		1
Yes	<0.001	17.1 (9.1 -29.6)	<0.001	7.8 (3.7 -16.4)
